# The Role of Polydimethylsiloxane in the Molecular Structure of Silica Xerogels Intended for Drug Carriers

**DOI:** 10.3797/scipharm.1409-08

**Published:** 2015-07-01

**Authors:** Katarzyna Czarnobaj

**Affiliations:** Department of Physical Chemistry, Medical University of Gdańsk, al. gen. J. Hallera 107, 80-416 Gdańsk, Poland

**Keywords:** Sol-gel method, Ormosils, Drug delivery systems

## Abstract

The aim of this study was to prepare and examine polymer/oxide xerogels with metronidazole (MT) as delivery systems for the local application of a drug to a bone. The nanoporous SiO_2_-CaO and PDMS-modified SiO_2_-CaO xerogel materials with different amounts of the polymer, polydimethylsiloxane (PDMS), were prepared by the sol-gel method.

Characterization assays comprised the analysis of the composite materials by using Fourier transform infrared spectroscopy (FTIR), determining the specific surface area of solids (BET), using X-ray powder diffraction (XRD) and scanning electron microscope (SEM) techniques, and further monitoring in the ultraviolet and visible light regions (UV-Vis) of the *in vitro* release of the drug (metronidazole) over time. According to these results, the bioactive character and chemical stability of PDMS-modified silica xerogels have been proven.

The release of MT from xerogels was strongly correlated with the composition of the matrix. In comparison with the pure oxide matrix, PDMS-modified matrices accelerated the release of the drug through its bigger pores, and additionally, on account of weaker interactions with the drug.

The obtained results for the xerogel composites suggest that the metronidazole-loaded xerogels could be promising candidates for formulations in local delivery systems particularly to bone.

## Introduction

The sol-gel-processed silica-based materials have received great attention due to their characteristics being favorable for medical studies focused on bone implants and carriers of drugs. These materials are non-toxic and biocompatible *in vivo*. Additionally, they possess the appropriate physicochemical properties: high surface area and volume of pores, good chemical and thermal stability, and they are bioactive.

The sol-gel process involves metal alkoxide or salt precursors, ethanol, and water. The hydrolysis and condensation of the liquid substrates leads to the formation of a colloidal dispersion – sol, which then undergoes crosslinking to form a three-dimensional solid oxide network – gel, and further, after drying – xerogel [1–3].

Sol-gel processing is classified as a technique which can produce an amorphous pure oxide xerogel (especially SiO_2_), or inorganically modified silicates with: CaO, P_2_O_5_, TiO_2_, ZrO_2_, and others. For a medical application, the introduction of CaO, P_2_O_5_, and TiO_2_ oxides has the greatest benefit. Mixed oxides simulate the mineral composition of bones, which also affect the bioactivity of the material (especially CaO which improves the bioactive properties of silica) [4–8].

One of the interesting features of sol-gel-processed oxides is the possibility of organic modifications that can be used to both adjust surface functionality and change textural properties of silica oxide. These organic-inorganic hybrid xerogels are defined as „Ormosils”; that is, organically modified silicates. The organic components are introduced into the liquid alkoxide where they are co-condensed with the molecules of hydrolyzed alkoxides. Over recent years [9–11], organically modified sol-gel-derived materials have been drawing much attention as a new kind of biomaterial which could be used for bone tissue substitution, due to their unique physical-chemical properties and bioactivity. Organic chains give structural flexibility by reducing the degree of crosslinking of the oxide network, thereby improving the mechanical performance of the finished material, and also changing the porosity of the material and thus could provide higher selectivity for specific controlled delivery. In comparison with hard and brittle oxide materials, the physical properties of the organic/inorganic composites are often much more closely related to biological structures.

In the present work, SiO_2_ and polydimethylsiloxane-modified SiO_2_ with different amounts of polymer within the oxide network have been successfully synthesized via the sol-gel route by using tetramethoxysilane (Si(OCH_3_)_4_) and hydroxyl-terminated polydimethylsiloxane (PDMS) as an organic modifier of the oxide network. This polymer, with a recognized position in tissue engineering, was selected due to its a unique combination of characteristics which include flexibility of the siloxane backbone, thermal and chemical stability, and hydrophobicity [12–16]. Additionally, calcium chloride as a precursor of Ca^2+^ ions was added in order to improve the bioactivity of the obtained materials.

The effect of the polymer on the structural, textural, and bioactive properties of the obtained materials was evaluated on the basis of the results of FTIR, BET, SEM, and XRD studies.

Also, this study examined the possibility of using PDMS-modified silica xerogels as the carrier materials for the controlled release of metronidazole, a drug with activity against anaerobic protozoa, aerobic, and microaerophilic bacteria, applied in periodontal disease treatment. Due to the weak blood supply of bone tissue and the poor penetration of drug to the bone, the implantable drug delivery seems to be a very attractive solution. The use of this form of drug will result in a shorter period of drug treatment of bone disease and the local release of the optimal drug concentration, which does not cause systemic toxic effects.

The drug was incorporated into the xerogels during the stage of the sol solution. Subsequently, the research aimed to determine the dynamics of metronidazole release from xerogels to the dissolution medium (SBF – simulated body fluid).

## Experimental

### Materials

Tetramethoxysilane (TMOS, C_4_H_12_O_4_Si), hydroxyl-terminated polydimethylsiloxane (PDMS, n=200, d=0.97), and calcium chloride (CaCl_2_) from Sigma Chemicals Company were used without further purification. Ethanol and ammonium hydroxide (from POCh Co., Poland) were of analytical grade purity.

Simulated body fluid (SBF) as the dissolution medium for the bioactivity test was prepared by dissolving reagent grade NaCl (136.8 mM), NaHCO_3_ (4.2 mM), KCl (3.0 mM), K_2_HPO_4_·3H_2_O (1.0 mM), MgCl_2_·6H_2_O (1.5 mM), CaCl_2_·2H_2_O (2.5 mM), and Na_2_SO_4_ (0.5 mM) (from POCh Co., Poland, analytical grade purity) in redistilled water and the solution was buffered with tris(hydroxymethyl)aminomethane [TRIZMA] (from Sigma) and hydrochloric acid to pH 7.40 [17].

### Sample Preparations

Tetramethoxysilane, calcium chloride, and ammonium hydroxide were used as starting inorganic constituents. Polydimethylsiloxane was used as the organic component. The molar ratio of TMOS, water, and CaCl_2_ was kept constant at 1:4:0.17. The ratio of PDMS to silica was kept at 30 and 50%. The catalyst used was 25% NH_3_·H_2_O (pH_sol_ = 9). Metronidazole (MT) was added into the precursor solution and continuously stirred until gelation. The gelation time for this sol was 15 min. The used wet gels were kept at 50°C for aging and drying for 3 days. The final materials were in the form of translucent, crack-free monoliths containing the drug in an amount of 0.4 mg/g xerogel.

The procedures of obtaining the different types of xerogels are given below.

SiO_2_-CaO: a solution of 2.53 g TMOS precursor in 5 mL MeOH, and a solution of 0.32 g of CaCl_2_ in 3 mL MeOH were separately stirred for 0.5 h. After that, the prepared solutions were mixed, and 1 mL water, 0.018 mL of 25% NH_3_·H_2_O, and 0.53 mg of metronidazole in 2 mL MeOH were added to form the homogenous gel.

SiO_2_-CaO-30%PDMS: a solution of 1.77 g TMOS precursor in 4 mL MeOH, a solution of 0.3 g PDMS in 4 mL MeOH, and a solution of 0.22 g of CaCl_2_ in 1 mL MeOH were separately stirred for 0.5 h. After that, the TMOS and PDMS prepared solutions were mixed for 0.5 h, and then CaCl_2_ solution, 1 mL water, and 0.016 mL of 25% NH_3_·H_2_O and 0.49 mg of metronidazole in 2 mL MeOH were added to form the homogenous gel.

SiO_2_-CaO-50%PDMS: a solution of 1.27 g TMOS precursor in 4 mL MeOH, a solution of 0.5 g PDMS in 4 mL MeOH, and a solution of 0.16 g of CaCl_2_ in 1 mL MeOH were separately stirred for 0.5 h. After that, the TMOS and PDMS prepared solutions were mixed for 0.5 h, and then CaCl_2_ solution, 1 mL water, and 0.016 mL of 25% NH_3_·H_2_O and 0.46 mg of metronidazole in 2 mL MeOH were added to form the homogenous gel.

### Characterizations

#### Textural Properties

The porosity of the samples was determined by surface area and average pore size measurements.

Specific surface area and pore size of the xerogels were measured using the BET technique based on nitrogen gas adsorption (Micromeritics ASAP 2405N apparatus). Before the measurement, the samples were crushed and degassed at 200°C. The specific surface area was calculated from the BET equation. An average pore size was calculated by the BJH method based on the desorption branch of the isotherm.

The morphology of xerogels was observed using a scanning electron microscope (Hitachi Scanning Electron Microscope (SEM) S-2500).

For SEM, the silica xerogel specimens were mounted on stubs and dried overnight in a vacuum desiccator. Prior to microscopy, the specimens were sputter coated with gold (Edwards, Model s150B Sputter Coater). Subsequently, the specimens were photographed and the microstructure of the silica xerogels was examined with a scanning electron microscope.

#### Molecular (Chemical) Structure

The chemical composition of silica xerogels was determined with the Jasco FT/IR-410 spectrometer. The spectra were measured over a range of 4000–400 cm^−1^ with an instrument resolution of 4 cm^−1^. Each individual spectrum was an average of 36 scans. In this measurement, the pulverized silica xerogels were mixed homogeneously with KBr powder and the background noise was corrected with pure KBr data.

Stability Tests

The chemical stability of the xerogels were monitored using the pH-meter Lab 860, SI Analytics GmbH, and the Conductometer Lab 960, SI analytics GmbH, by measuring the pH and conductivity of the SBF solution in which the xerogel samples were incubated in a water bath at 37 ± 0.1°C for the period of 3 months.

Bioactivity Test

The bioactive potential of the xerogels was tested by using the *in vitro* Kokubo test [18]. The simulated body fluid (SBF) that has inorganic ion concentrations similar to those of human extracellular fluid was used in order to reproduce the formation of apatite on bioactive materials *in vitro*. A high concentration of silanols (Si-OH) on the oxide surface, the presence of Ca^2+^ in the oxide network, and the presence of Ca^2+^, HPO_4_^2-^, and HCO_3_^-^ ions in the SBF solution have been shown to stimulate the formation of an amorphous calcium phosphate layer and then the hydroxyapatite nanocrystals (HA) [19].

Samples of xerogels were incubated in SBF at 37°C for 30 days (0.5 g / 50 mL). After incubation, the xerogels were dried at 80°C for 1 day and examined using FTIR and XRD techniques (Philips X-Pert diffractometer) to determine the ability to form an apatite layer.

#### Release Studies

The drug release process was evaluated via diffusion of MT from the pores of the xerogel materials.

Prior to the release studies, the resulting monolithic xerogels were crushed and sieved to a desired diameter of granules 1.6–2 mm. The xerogel samples (0.5 g) were exposed to releasing testing in 50 mL of simulated body fluid (SBF) as the dissolution medium at 37°C (±0.5°C) under the sink condition of the drug (the maximum working concentration of the drug in the dissolution medium was well below the 10% saturated solution of a drug substance, which is a requirement for fulfillment of ‘sink’ conditions), using the thermostated shaking water bath (Julaba, 50 spm). The concentration of MT in SBF was measured by the proposed spectrophotometric method (Hewlett Packard 8452A UV–Vis spectrophotometer, λ = 320 nm) by taking 2 mL of the solution at selected intervals based on drug release. The release medium was replaced by a fresh SBF solution (2 mL) in order to maintain a volume of 50 mL. The amount of MT obtained from the drug release studies was calculated from a linear regression equation.

## Results and Discussion

### Chemical Structure and Morphology of Composite Xerogels

**Fig. 1:**
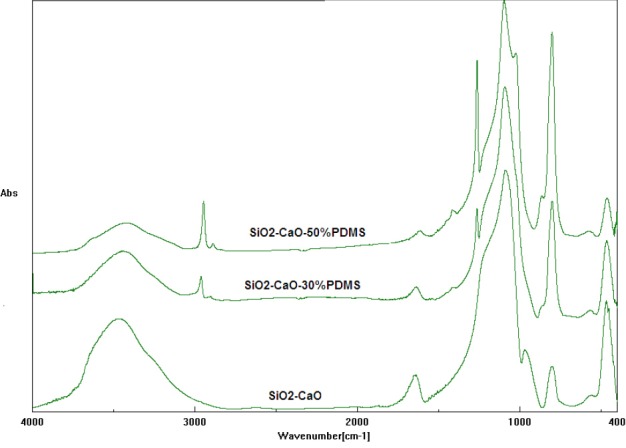
FTIR spectra of SiO_2_-CaO, SiO_2_-CaO-30%PDMS, and SiO_2_-CaO-50%PDMS xerogels

The obtained xerogels were characterized using the FTIR technique as illustrated in Figure 1. The FTIR analysis showed that the oxide xerogel was completely hydrolyzed and well polymerized. The observed strong bands were caused by the vibrations of Si-O-Si groups at 1076 cm^−1^, 924 cm^−1^, and 800 cm^−1^ indicative of a high degree of polymerization. The lack of bands derived from the CH stretching vibrations in the region of 2800 cm^−1^ indicates complete hydrolysis of the alkoxy groups (-OCH_3_,) derived from tetramethoxysilane. Two broad bands, one at 3450 cm^−1^ and the other at 1631 cm^−1^, were caused by O-H vibrations of Si-OH groups and the water molecule retained in the pores of the oxide network. Bands belonging to CaO, occurring in the region of 1700–1460 cm^−1^ were masked or occurred together with strong bands assigned to the vibration of water molecules [20].

The FTIR spectra of the oxide xerogels modified with PDMS were slightly different from the spectrum of the pure oxide xerogel. Additional peaks at 2800, 1260, and 795 cm^−1^ were observed when PDMS was used for the sample preparation. The band at 2800 cm^−1^ was assigned to the fundamental stretching vibrations of C-H bonds. The bands at 1260 and 795 cm^−1^ were due to the bending vibrations of Si-CH_3_ bonds belonging to PDMS [21]. Significant differences existed for the intensity of the band in the range 4000–2000 cm^−1^ for the samples with PDMS and without PDMS. The intensity of the bond responsible for oscillations in the O-H group was lower for the silica xerogels that were modified PDMS. This is because of the hydrophobic nature of PDMS that reduced the O-H group and the water molecules inside of the xerogel lattices.

The evaluations of the xerogels’ textural properties were conducted by the BET technique by nitrogen multilayer adsorption measured as a function of relative pressure.

**Fig. 2:**
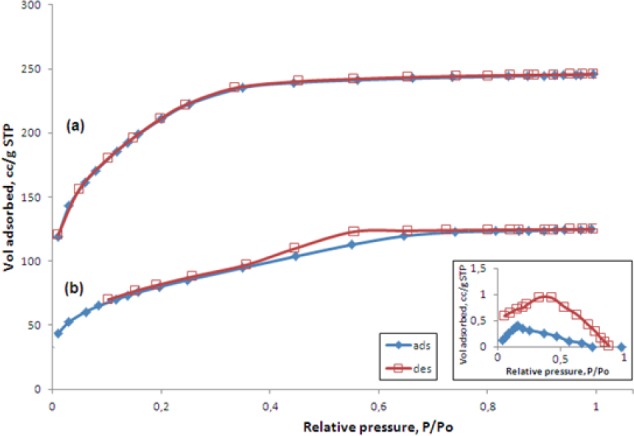
Nitrogen adsorption-desorption isotherms of xerogels: SiO_2_-CaO (a), and SiO_2_-CaO-30%PDMS (b), (insert map: BET isoterms of SiO_2_-CaO-50%PDMS xerogel)

As shown in Figure 2, the SiO_2_-CaO xerogel reveals a type I isotherm with a sharp initial increase of the adsorbed gas volume at a low relative pressure p/p_o_, indicating that the xerogel has a significant contribution of micropores (pore diameter <2 nm), according to the IUPAC recommendation [22, 23].

The isotherm for the SiO_2_-CaO-30%PDMS sample is a type IV. This isotherm shows low adsorption at low relative pressures and a sudden increase in adsorption occurs at high pressure, which is characteristic of a mesoporous solid (pore radii ≥ 2 nm). The occurrence of hysteresis in the desorption branch is attributed to the presence of pore cavities larger in diameter than the openings leading into them. The isotherm for the SiO_2_-CaO-50%PDMS (insert in Fig. 2) indicates that this xerogel is characterized by very small values of adsorption, resulting from the very small surface area. These results prove that this material is macroporous, practically non-porous.

As presented in Table 1, PDMS addition to the oxide xerogel changes porosity parameters. For SiO_2_-CaO and PDMS-modified xerogels, the differences between pore diameters, pore volumes, and surface area values were found to be highly significant. For SiO_2_-CaO-50%PDMS, the amount of introduced PDMS resulted in nearly complete filling of pores. A large decrease in the specific surface area (from 752, through 252 to 1.74 m^2^g^−1^) and total pore volume (from 0.253 through 0.21 to 0.0013 cm^3^g^−1^ with an increase in PDMS content (0%, 30%, 50%, respectively)) indicate decreased porosities of the samples. This effect is associated with the formation of interpenetrating networks between the polymer and porous oxide phases.

**Tab. 1:**
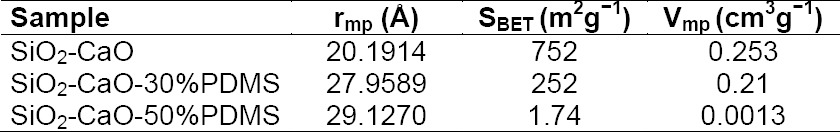
Some physical properties of the obtained xerogels: pore size (r_mp_), surface area (S_BET_), and pore volume (V_mp_)

**Fig. 3:**
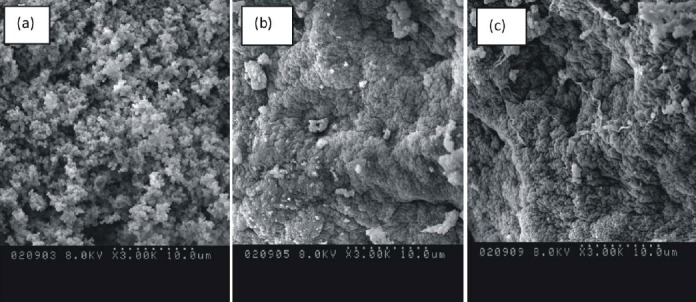
SEM micrographs for SiO_2_-CaO (a), SiO_2_-CaO-30%PDMS (b), and SiO_2_-CaO-50%PDMS (c) xerogels

The morphologies of the xerogels with different amounts of PDMS were characterized using SEM (Fig. 3). It is clearly observed that the morphology of the samples changes from a globular structure for non-modified xerogel to a dense morphology for samples with PDMS as an additive. Xerogels without the addition of PDMS have a structure with several smaller agglomerates joining together to form a larger cluster, while the xerogels with PDMS have an unbranched and less convoluted structure.

#### Stability Tests

**Fig. 4:**
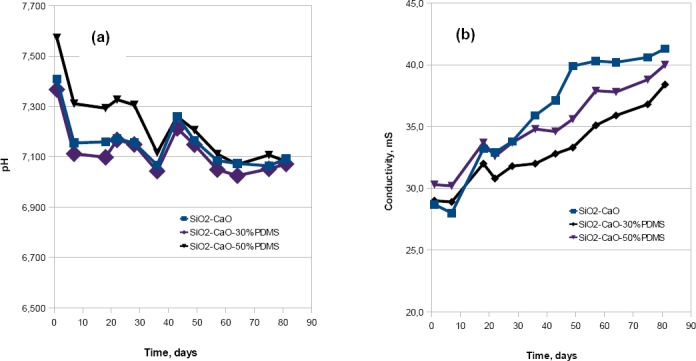
The chemical stability of the xerogels: (a) pH, and (b) conductivity of the SBF in which the xerogel samples were incubated for a period of three months

The chemical stability of the xerogels was assessed by examining the change in pH and conductivity of the SBF in which the xerogel samples were incubated. Each sample was incubated for a period of three months. For each SBF solution, a slight variation in pH value was observed over time, ranging from 0.02 to 0.025 pH units (Fig. 4a).

The conductivity of each SBF solution slightly increased with increased incubation time (Fig. 4b). These changes ranged from 8 to 13 mS, for the value of conductivity of about 30 mS. This can be explained by the slow release rate of silicic acid and Ca^2+^ ions from the matrix.

The lack of significant changes in pH and conductivity of the SBF indicates that the obtained xerogels are chemically stable.

#### Bioactivity Test

The bioactive potential of xerogels was tested by using the *in vitro* Kokubo test. Samples of xerogels were incubated in SBF at 37°C for 30 days (0.5 g / 50 mL). After incubation, the xerogels were dried at 80°C for 1 day and examined using FTIR and XRD techniques, to determine the ability to form an apatite (HA) layer.

**Fig. 5:**
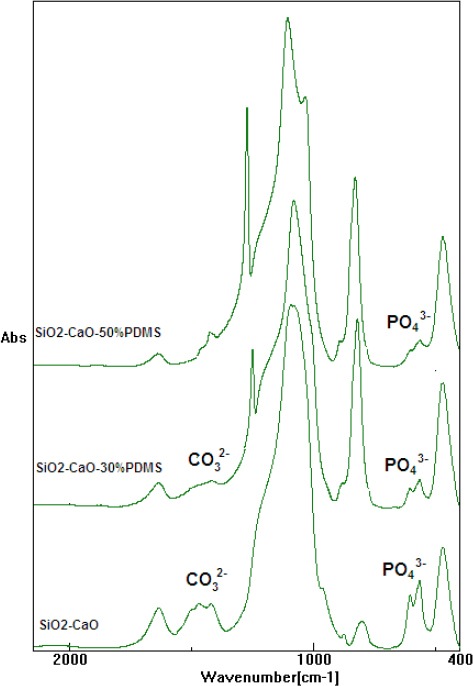
FTIR spectra of all xerogel samples after soaking in SBF solution for 1 month

The FTIR spectra for all xerogels showed two new bands at about 550 and 600 cm^−1^ wavenumbers (the major absorption mode of the phosphate groups, the O–P–O bending mode), which indicate the formation of crystalline apatite (Fig. 5). Before incubation in the SBF, the FTIR spectra of the tested xerogels showed a single, weak band observed at about 580 cm^−1^ (vibration of Si-O). However, following longer incubation of the xerogels in the SBF solution, a sharp doublet appeared at 564 and 602 cm^−1^, indicating the formation of crystalline hydroxyapatite. Additionally, carbonate groups substituting the PO_4_^3-^ ions in the apatite structure can be detected in all xerogels by the appearance of bands at 1410–1460 cm^−1^ [24, 25].

The induction period for the crystallinity of HA depends on the composition of the xerogels. It was observed that the increase in the percentage of PDMS in xerogels results in slower growth of hydroxyapatite on the surface of the composites. The differences in the HA growth on the xerogel surface are related to its formation mechanism. The OH groups from the oxide xerogels form hydrogen bonds with the components of the SBF solution, especially with Ca^2+^, CO_3_^2-^, and HPO_4_^2-^ ions, resulting in the growth of HA on the surface of the xerogels. In the case of xerogels with PDMS, hydrophobic groups in the structure of PDMS and a lower amount of silanol groups probably retards the crystallization of hydroxyapatite.

To confirm the ability to grow apatite on the surface of materials, the XRD test was performed.

**Fig. 6:**
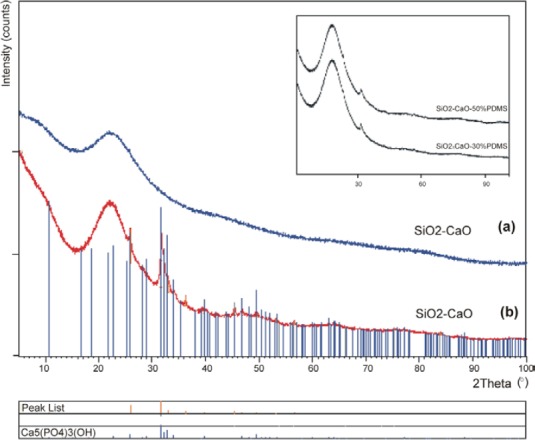
The XRD patterns of the SiO_2_-CaO xerogel before (a), and after (b) 1 month of soaking in SBF solution (insert map: XRD patterns of SiO_2_-CaO-PDMS xerogels)

The X-ray diffraction profiles of the obtained xerogels are shown in Figure 6. At the beginning of the experiment, the samples exhibited no diffraction peaks and a high background (“halos”) intensity in the range of 20–30° 2θ, indicating that they are highly disordered, amorphous materials (diffraction profile “a” for SiO_2_-CaO xerogel). After immersion in the SBF, the X-ray diffraction analysis showed that all xerogels produced new peaks demonstrating the surface crystallization (e.g. diffraction profile “b” for SiO_2_-CaO xerogel). The most intense peaks occurred at 2θ 32°, 26° which are 2 1 1 and 0 0 2 diffractions, respectively, of the apatite (according to the standard JCPDS cards (09-0432)) [26]. As apparent from the diffraction pattern, the content of the crystalline phase decreases for samples with PDMS in the oxide matrix (insert in Fig. 6).

#### Drug Release Analysis

**Fig. 7:**
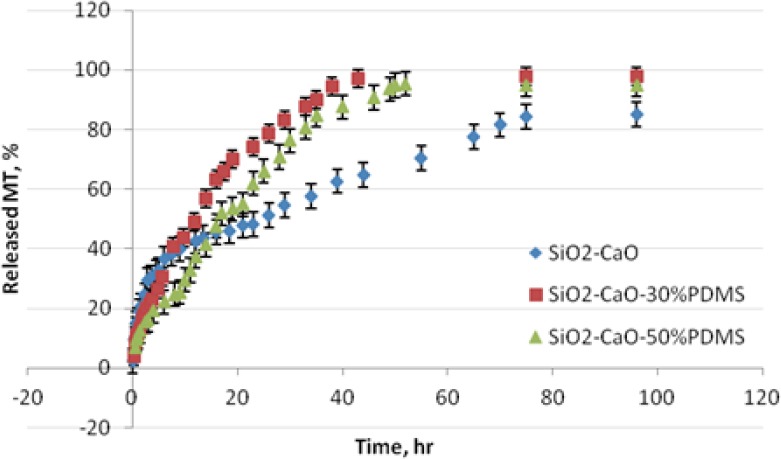
The release profiles of MT from SiO_2_-CaO, SiO_2_-CaO-30% PDMS, and SiO_2_-CaO-50% PDMS xerogel carriers

The release tests were followed *in vitro* in SBF solution for 5 days. Figure 7 displays the plot of the data expressed as the cumulative amount of metronidazole released from the xerogel matrices as a function of time. The formulations displayed visible different release profiles. MT was released from the SiO_2_-CaO matrix in a biphasic way characterized by a faster initial release (about 3 h) followed by a slower, steady release. The PDMS-modified silica xerogels released the drug in a more monotonous, linear course, without the apparent initial burst of MT.

This study shows that the relative proportion of SiO_2_ and PDMS determines the release rate of the drug. When comparing the drug release rates from different matrices, a slower release rate was observed in the pure oxide matrix (without PDMS). This study showed that 50% of the drug was released within 25 hr from non-modified oxide, and 12 and 17 hr from xerogels containing 30% and 50% PDMS, respectively. Release profiles of MT from SiO_2_-CaO and SiO_2_-CaO-PDMS did not converge to a common value. The maximum amount of MT that was released from the SiO_2_-CaO, SiO_2_-CaO-30% PDMS and SiO_2_-CaO-50% PDMS varied from 86, 95, to 98% of the nominal loading, respectively. The release was over after 75 hr for pure oxide, 43 hr for 30% PDMS-modified matrix and 52 hr for 50% PDMS-modified matrix.

One of the reasons affecting the drug release rate may be the difference in porosity of the oxide and PDMS-modified oxide matrices. The SiO_2_-CaO xerogel is microporous in comparison with mesoporous SiO_2_-CaO-30% PDMS, so the lower release rate of the drug may be attributed to its decreased pore size. During the first 3 hours of release, the release proceeds mainly by dissolution and diffusion of the drug in the superficial layers of the xerogel. Therefore, metronidazole was released quickly at first, in spite of small porosity. Within the inner part of the xerogel, the microporous network of the matrix effectively immobilized the metronidazole molecules, making it difficult for the drug to pass to the surrounding fluid. Mesoporous SiO_2_-CaO-30% PDMS, due to larger pores, makes it easier to release the drug. SiO_2_-CaO-50% PDMS xerogel differs greatly in porosity parameters in comparison with the SiO_2_-CaO-30% PDMS one. In spite of similar pore sizes (27 - 29 Å), it was characterized both by a minimal surface area and pore volume, therefore, lower porosity slows down the release of the drug.

Moreover, another important reason for the various drug release rates is the polarity of the xerogel surface, which will influence the interactions between the drug substance and the matrix. It has been shown in previous studies [27] that the hydrogen bonds between the molecules of metronidazole and silanol groups of oxide xerogels exist. The pure oxide xerogels have a lot of hydroxyl groups on the surface of the pores through which they can interact (through hydrogen bonds) with the drug and thus slow down its release. The methyl groups present on the surface of the PDMS-modified oxide decrease the hydrophilicity/polarity of silica, by reducing the amount of OH groups (confirmed by the FTIR study). -CH_3_ groups cannot interact electrostatically with the hydrophilic drug molecules, and this might be the additional cause of a slightly faster release of MT.

In summary, PDMS-modified matrices accelerate the release of the drug through its bigger pores, and additionally, on account of weaker interactions with the drug.

## Conclusion

The oxide materials (such as glasses, xerogels, or ceramics) are exceptional due to their bioactive properties, the ability to bond to living bone through the apatite layer formed on their surfaces in the body. The organic modification of such materials may additionally improve their mechanical features, especially tame their brittle characteristics. It is expected that organic modification of the oxide components would produce novel biocompatible biomaterials with both high bioactivity and high flexibility.

The non-degradable polydimethylsiloxane (PDMS), biocompatible, noncarcinogenic, nontoxic, and bio-stable in the human body, is one of the attractive polymers which can be considered as a component in bone-substituting composites.

In this study, the sol-gel-prepared SiO_2_-CaO and PDMS-modified SiO_2_-CaO xerogels were used as carrier matrices. The aim of the work was to investigate how the organic polymer, PDMS in the oxide network, influences the microstructure, stability, and bioactivity of the composite matrix.

The conducted experiments proved that the obtained xerogels are bioactive *in vitro* and chemically stable in simulated body fluid.

The sol-gel method was also found to be useful for the synthesis of efficient drug carriers for controlled release of metronidazole. This research showed that carrier modification by co-condensation of organic species (PDMS) during synthesis permits the creation of new surface properties, and thus could provide higher selectivity for specific controlled delivery. The release of MT from xerogels was strongly correlated with the composition of the matrix. With increasing concentrations of PDMS, the initial drug burst was reduced. Additionally, in comparison with the pure oxide matrix, PDMS-modified matrices accelerate the release of the drug through its bigger pores, and on account of weaker interactions with the drug.

Based on these results, PDMS-modified silica-based xerogels could be attractive candidates as the bone-substitute materials and as polymeric-oxide carriers for local controlled drug delivery.
